# G-CSF and G-CSFR Induce a Pro-Tumorigenic Macrophage Phenotype to Promote Colon and Pancreas Tumor Growth

**DOI:** 10.3390/cancers12102868

**Published:** 2020-10-06

**Authors:** Ioannis Karagiannidis, Eliane de Santana Van Vilet, Erika Said Abu Egal, Brandon Phinney, Damian Jacenik, Eric R. Prossnitz, Ellen J. Beswick

**Affiliations:** 1 Division of Gastroenterology, Department of Internal Medicine, University of Utah, Salt Lake City, UT 84132, USA; yannis.karagiannidis@hsc.utah.edu (I.K.); u6015847@utah.edu (E.d.S.V.V.); Erika.Egal@hsc.utah.edu (E.S.A.E.); 2 Division of Molecular Medicine, Department of Internal Medicine; Autophagy, Inflammation and Metabolism Center of Biomedical Research Excellence, University of New Mexico Comprehensive Cancer Center; University of New Mexico Health Sciences Center, Albuquerque, NM 87131, USA; BPhinney@salud.unm.edu (B.P.); damian.jacenik@biol.uni.lodz.pl (D.J.); eprossnitz@salud.unm.edu (E.R.P.); 3 Department of Cytobiochemistry, Faculty of Biology and Environmental Protection, University of Lodz, 90-236 Lodz, Poland

**Keywords:** G-CSF, G-CSFR, TAMs, tumor microenvironment

## Abstract

**Simple Summary:**

The gastrointestinal tumor microenvironment is regulated by cytokine production from the tumor and adjacent immune cells. G-CSFR is a cytokine that is highly produced in the gastrointestinal tumor microenvironment and may have unrecognized pro-tumorigenic activities. Here, we explored the impact of G-CSF and its receptor on macrophage responses in colon and pancreas tumors in mice. We found G-CSF/G-CSFR to promote a pro-tumorigenic macrophage response. Upon deletion of G-CSFR, macrophages exhibited increased tumor killing ability in culture and decreased tumor growth in mice. Our findings suggest that G-CSFR blockade may be beneficial to promoting anti-tumorigenic macrophage responses and should be further examined as a therapeutic target.

**Abstract:**

Tumor-associated macrophages (TAMs) in the gastrointestinal tumor microenvironment (TME) are known to polarize into populations exhibiting pro- or anti-tumoral activity in response to stimuli such as growth factors and cytokines. Our previous work has recognized granulocyte colony-stimulating factor (G-CSF) as a cytokine capable of influencing immune cells of the TME exhibiting pro-tumoral activity. Here, we aimed to focus on how G-CSF regulates TAM phenotype and function and the effects on gastrointestinal (GI) tumor progression. Thus, wildtype (WT) and G-CSFR^−/−^ macrophages were examined for cytokine production, gene expression, and transcription factor activity. Adoptive transfer of WT or G-CSFR^−/−^ macrophages into tumor-bearing mice was performed to study their influence in the progression of colon (MC38) and pancreatic (PK5L1940) tumor mouse models. Finally, the difference in cytotoxic potential between WT and G-CSFR^−/−^ macrophages was examined both in vitro and in vivo. Our results indicate that G-CSF promotes increased IL-10 production and decreased IL-12 production, which was reversed in G-CSFR^−/−^ macrophages for a pro-inflammatory phenotype. Furthermore, G-CSFR^−/−^ macrophages were characterized by higher levels of NOS2 expression and NO production, which led to greater tumor related cytotoxicity both in vitro and in vivo. Our results suggest that in the absence of G-CSFR, macrophage-related tumor cytotoxicity was amplified. These findings, along with our previous reports, pinpoint G-CSF /G-CSFR as a prominent target for possible clinical applications that aim to control the TME and the GI tumor progression.

## 1. Introduction

Gastrointestinal cancers, and more specifically pancreatic and colorectal cancer (CRC), are aggressive malignant diseases with a low survival rate and poor prognosis for late stage disease. Colon cancer is the third most common cancer worldwide with grave impacts on global health. To date, causes of GI cancer have been linked not only to environmental, nutritional, and genetic/epigenetic factors, but also to age and chronic inflammation diseases [1]. Within GI tumors, chronic inflammatory conditions are usually present with several immune cells having been identified in the tumor microenvironment (TME), including T cells (both cytotoxic and helper cells), mesenchymal stem cells, and dendritic cells, as well as tumor-associated macrophages (TAMs) [2,3]. TAMs originate from a monocytic lineage and are recruited to the tumor site through chemokines such as CCL2, vascular endothelial growth factor (VEGF), CCL5, and TGF-β [4]. TAMs are highly infiltrative and have been identified in high numbers in the CRC and pancreatic cancer TME [5,6]. During tumor progression, TAMs are not terminally differentiated, but present with plasticity with the ability to polarize into distinct directions of inflammatory macrophages (tumor-resistant cells) or anti-inflammatory macrophages (sometimes referred to as M1 and M2, respectively) [7]. M1 macrophages, also described as classically activated, are polarized by microenvironment signals such as interferon-gamma (IFN-γ) promoting signal transducer and activator of transcription 1 (STAT1) signaling and lipopolysaccharide (LPS) activation of toll-like receptors (TLRs) [8]. Upon TLR activation, M1 cells produce pro-inflammatory cytokines such as TNF-α, IL-12, and IL-6, which may promote tumor cell killing through NOS2 production and activation of T cells [9,10]. Polarization of M2 macrophages is mediated through STAT3 and STAT6 pathways and causes production of several anti-inflammatory cytokines such as IL-10, IL-13, IL-4, and TGF-β, but also arginase-1 (ARG1), mannose receptor (MR, CD206), and scavenger receptors CD163 [11,12]. These cytokines have been reported to play a crucial role in the TME of pancreatic and colon cancers [13,14]. 

One cytokine that has not been well examined in macrophage function is granulocyte colony-stimulating factor (G-CSF). G-CSF regulates the differentiation and activation of neutrophils and may play a key role in regulating cytokine responses [15,16]. Our group has previously shown that G-CSF and its receptor are highly expressed in human and mouse GI cancers [17,18]. Treatment with anti-G-CSF antibodies led to decreased tumor growth and elevated numbers of tumor immune cells by promoting protective anti-tumor immunity. A pro-tumorigenic function of G-CSF in breast cancer has been observed with G-CSF promoting breast cancer cell migration and an anti-inflammatory macrophage phenotype [19]. 

Taking into account the above observations, in this study, we set out to elucidate how G-CSF affects the gastrointestinal tumor microenvironment through the regulation of macrophage phenotype and function. Our experiments revealed that G-CSFR^−/−^ macrophages presented with a cytokine profile tending towards the M1-like phenotype with altered STAT signaling. G-CSFR^−/−^ macrophages demonstrated increased NOS2 expression and tumor killing ability, while the addition of G-CSF to wildtype (WT) macrophages led to decreased NOS2 activity and increased IL-10 production to promote a pro-tumorigenic phenotype. Adoptive transfer of G-CSFR^−/−^ into colon or pancreas tumor-bearing mice led to decreased tumor growth and increased NOS2 and apoptosis. Overall, our results demonstrate that, in the presence of G-CSF/G-CSFR, macrophages exhibit a pro-tumorigenic phenotype in the TME of the colon and pancreatic cancer.

## 2. Material and Methods

### 2.1. Mouse Models

C57BL/6 WT mice were purchased from the Jackson Laboratory. C57/BL/6 G-CSFR^−/−^ mice were obtained from Daniel Link (Washington University School of Medicine) and were backcrossed with WT mice. The animals were housed in a pathogen-free facility at constant temperature (22–24 °C), relative humidity ~55%, and maintained under 12-h light/dark cycle with access to standard chow pellets and tap water ad libitum. All animals used in this study had an average weight of 22 g (range, 19–25 g) prior to initiation of the experiments. This research has been approved by the University of New Mexico Health Sciences Center IACUC on 11/16/16 (protocol# 16-200595 and the University of Utah Health IACUC on 10/25/18 (protocol# 18-10004).

### 2.2. Tumor Cells

MC38 cells were obtained from the National Institutes of Health (NIH) and PK5L1940 cells were kindly provided by Dr. Michael Gough from the Earle A. Chiles Research Institute [20]. Cells were cultured in complete RPMI with 10% FBS, 1% penicillin/streptomycin, and 1% L-glutamine. Cells (2 × 10^6^) and 100 µL (PBS mixed 1:1 in Matrigel^®^, Corning, Tewksbury, MA, USA) were injected into the flank of C57B/L6 mice (6–8-week-old males or females). Tumors were measured using calipers throughout the studies, dissected on day 21, and divided for cell culture and RNA work.

### 2.3. Bone Marrow-Derived Macrophages

Bone marrow-derived macrophages (BMMs) were isolated from approximately 10 week old WT and G-CSFR^−/−^ mice. Briefly, femur bones were flushed with DMEM under sterile conditions, separately for each mouse. Isolated cells were pelleted and resuspended in 30 mL differentiation medium (Dulbecco’s modified eagle’s medium supplemented with 10% fetal bovine serum, 2 mM L-Glutamine, 30% L929 conditioned medium) as previously described [21]. The cells were incubated for seven days under 5% CO_2_ at 37 °C in order to differentiate towards the macrophage cell lineage. Differentiated BMMs cells were used for in vitro or in vivo experiments once their cell identity was reconfirmed by flow cytometry for the expression of CD11b and F4/80 macrophage-specific surface markers. Cells were plated at 2 × 10^5^ per well in 48-well culture dishes, activated with combinations of 1µg/mL LPS (Enzo Life Sciences, Farmingdale, NY, USA) and 100 μg/mL of IFNγ (Shenandoah Biotechnology, Warwick, PA, USA) and incubated under 5% CO_2_ at 37 °C for at least 24 h prior to experimental treatments. 

Supernatants were collected for cytokine analysis and cells were used for flow cytometry or qRT-PCR analysis. For nitrite/nitrate assays (Measure-IT colorimetric kit from ThermoFisher Scientific, Waltham, MA, USA), cells were incubated with 1 µg/mL of LPS and 100 μg/mL of IFNγ for 24 h and performed in accordance with the manufacturer’s instructions. Cultured BMMs were used for adoptive transfer into WT mice where 1 × 10^6^ cells were injected peritumorally on days 1 and 8.

### 2.4. Multiplex Cytokine Arrays

MC38 and P5KL1940 tumors were dissected into 8 mg pieces (± 0.5 mg) and incubated in RPMI complete medium for 16 hours in an approach we have previously published [22,23]. Tumor culture or BMM supernatants were analyzed for cytokine and chemokine expression by multiplex array (MilliporeSigma, Burlington, MA, USA) in accordance with the manufacturer’s instructions.

### 2.5. Flow Cytometry

Flow cytometry was used to identify macrophage phenotype markers related to anti- or pro-tumor activity as well as cytotoxicity. In vitro differentiated BMMs were washed using flow cytometry buffer (PBS with 1% BSA) and pelleted by centrifugation at 300× *g* for 10 min. The supernatant was removed, pellets resuspended in fixation and permeabilization solution using 2% paraformaldehyde in PBS, and incubated for 45 min on ice. Subsequently, cells were washed as described above and incubated with anti-mouse antibodies for NOS2 (clone CXNFT), Arg1 (clone A1exF5), or isotype controls (all from eBioscience, San Diego, CA) for 45 min at 4 °C in the dark. For surface markers, cells were stained with primary antibodies for CD11b (clone M1/70), MHC-II (clone M5/114.15.2), CD115 (clone AFS98), and GR-1 (clone RB6-8C5) (all from eBioscience) prior to fixation. Samples were stained in triplicate in order to serve as technical replicates. Compensation controls were prepared each time alongside the samples using UltraComp ebeads (Invitrogen, Carlsbad, CA, USA) and following the exact same staining process as the cells. Single color controls and FMO samples were prepared each time to eliminate autofluorescence and fluorochrome spillover. After washing twice, data were acquired using an Attune NtX flow cytometer (Invitrogen) and analyzed with Attune NtX software using FSC-SSC parameters to exclude debris and duplicates.

### 2.6. Quantitative Real-Time PCR

Tissue fragments or BMMs were homogenized in Trizol and RNA extraction was performed according to the manufacturer’s instructions. RNA was measured using a nanodrop (ThermoFisher Scientific, Waltham, MA, USA). Total RNA (100 ng/µL) was reversed transcribed using qScript^®^ cDNA SuperMix reverse transcription mix (Quantabio) with the following PCR settings: 25 °C for 5 min, 42 °C for 30 min, and 85 °C for 5 min. Quantitation of mRNA was performed using real-time PCR with validated FAM dye-labeled TaqMan^®^ probes (Applied Biosystems, Foster City, CA, USA) for IL-10, IL-12, ARG1, NOS2, BAD, CASP3, FASL, and BCL2. The reaction mixture consisted of cDNA, TaqMan Fast Advanced Master Mix (Applied Biosystems, Foster City, CA, USA), TaqMan^®^ Assays, and RNase-free water in a total volume of 10 μL. Cycle parameters for TaqMan^®^ assays were as follows: Initial denaturation at 95 °C for 3 min, followed by 50 cycles of sequential incubations at 95 °C for 15 s and 60 °C for 1 min. Results were normalized to the expression of *ACTB* (β-actin) using replicates for each sample. Real-time PCR was performed on Applied Biosystem’s Quant Studio 3 instrument (ThermoFisher Scientific, Waltham, MA, USA). The CT value, determined as the PCR cycle number that crossed the signal threshold, was used to analyze data using the comparative CT method (Sequence Detector User Bulletin 2; Applied Biosystems, Foster City, CA, USA) and reported as the fold change relative to the mRNA of the mouse housekeeping gene, *ACTB*.

### 2.7. Statistical Analysis

Results are presented as the mean ± standard error of mean (SEM). Data were analyzed using one-way ANOVA in GraphPad Prism 5 (San Diego, CA, USA). Values of *p* < 0.05 were considered statistically significant. The values regarding the quantitative differences in qPCR were expressed as the standard deviation (± SD). Five animals per mouse group were used with two experiments in both male and female mice. All in vitro experiments were run with replicates in a minimum of three separate experiments.

## 3. Results

### 3.1. G-CSFR^−/−^ BMMs Display an Altered Phenotype Compared to WT BMMs

**Figure 1:**
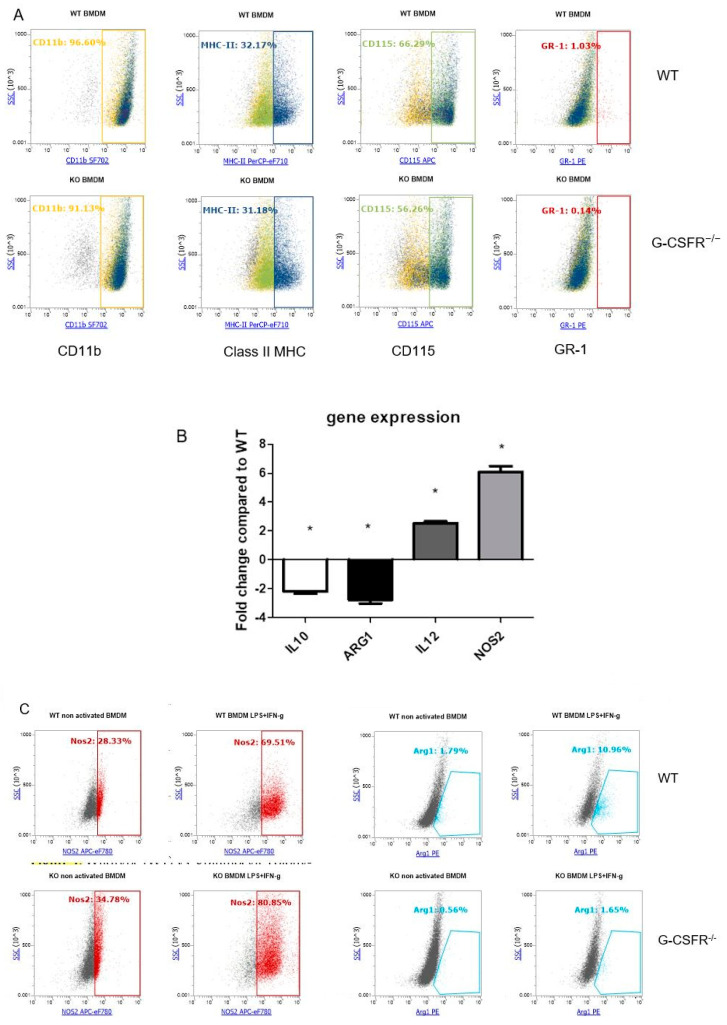
WT vs G-CSFR^−/−^ BMM express similar macrophage markers, but altered cytokine and NOS2 expression as shown by (**A**) Flow cytometry analysis for CD11b, MHC-II, CD115 and GR-1 and (**B**) Real-Time PCR analysis showing in the absence G-CSFR, there is downregulation of *IL-10* and *ARG-1* and upregulation of *IL-12* and *NOS2* mRNA levels compared to WT. (**C**) Results for *ARG-1* and *NOS2* were confirmed by flow cytometry showing increased NOS2 and reduced ARG-1 in LPS + IFNγ activated G-CSFR^−/−^ BMMs compared to WT. *n* = 6, * *p* < 0.05.

In order to study the impact of G-CSF/G-CSFR on macrophages, we compared WT vs. G-CSFR^−/−^ BMMs by flow cytometry and quantitative RT PCR (qRT PCR). To first test for possible global changes in the phenotype of differentiated macrophage, we examined commonly used macrophage/monocyte markers such as CD11b, MHC-II, CD115, and GR-1 (Figure 1A). Analyses revealed that there were no major differences between WT and G-CSFR^−/−^ BMMs. We also found that over 98% of the cultured cells were expressing F4/80 for both WT and G-CSFR^−/−^ BMMs (Figure S1A). For WT BMM, we also found that the CD115^-^ population was also G-CSFR^+^, and upon activation with LPS, CD115 expression was lost (Figure S1B). Next, we examined specific genes and proteins that have been associated with macrophage polarization showing pro- or anti-tumoral activity. Primary markers associated with M1 macrophages are NOS2 and IL-12 and for M2 macrophages, ARG-1 and IL-10. Other markers of M2 macrophages, CD163 and CD206, were not expressed by these cells. Our analysis demonstrated that in the absence of G-CSFR in BMM, IL-10 and ARG-1 gene expression were decreased compared to WT BMMs (Figure 1B). Opposing results were observed for IL-12 and NOS2 as markers of anti-tumorigenic macrophages, with 2- and 6-fold increases, respectively, in the absence of G-CSFR. The NOS2 and ARG-1 results were confirmed using flow cytometry, where NOS2 was increased in G-CSFR^−/−^ cells co-stimulated with LPS and IFNγ, while ARG-1 levels were higher in activated WT BMMs (Figure 1C). Taken together, these data indicate a direct effect of G-CSFR deficiency in enhancing anti-tumoral activity of BMMs.

### 3.2. G-CSF and G-CSFR Modulate Cytokine Production by Macrophages

**Figure 2:**
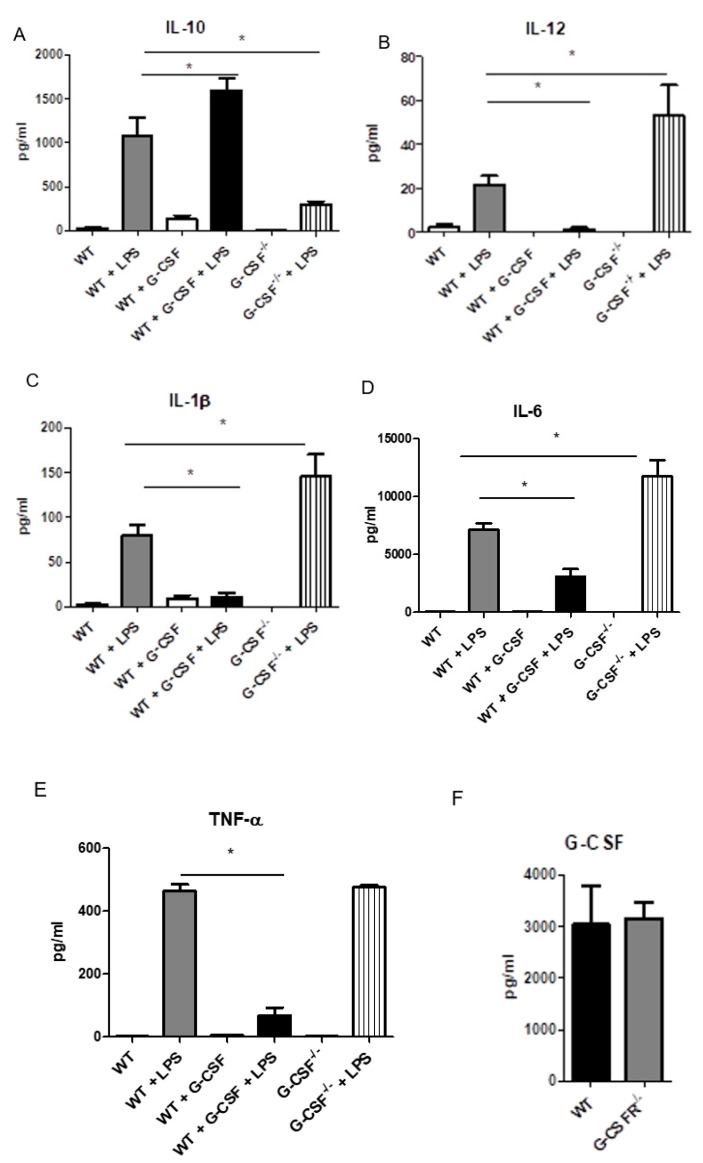
WT, G-CSFR^−/−^ or WT + G-CSF BMM activated by LPS produce different patterns of cytokines by multiplex array analysis of (**A**) IL-10 (**B**) IL-12, (**C**) IL1β (**D**), IL-6 and (**E**) TNF-α and (**F**) G-CSF, *n* = 8, * *p* ≤ 0.05.

Based on the previous results and in order to further investigate the enhancement in the anti-tumoral activity of G-CSFR^−/−^ macrophages, we sought to analyze in vitro cytokine production of activated BMMs. Multiplex cytokine array analysis revealed that IL-10 production was increased upon the activation of BMMs (Figure 2A). Furthermore, IL-10 production was increased upon treatment of WT cells with recombinant G-CSF. G-CSFR^−/−^ BMMs showed substantially lower levels of IL-10 production, suggesting a direct effect of G-CSF in IL-10 expression. We next analyzed the production of proinflammatory cytokines IL-12, IL-6, IL-1β, and TNF-α. Our results demonstrated that all these cytokines were significantly increased in G-CSFR^−/−^ compared to WT BMMs (Figure 2B–E). We also observed that co-activation of LPS-treated BMMs with G-CSF significantly reduced the levels of IL-12, IL-1β, Il-6, and TNF-α production in WT BMMs, indicating a role for G-CSF in opposing an anti-tumoral macrophage phenotype. In Figure 2F, WT C57BL/6 and G-CSFR^−^**^/^**^−^ BMM produce equal amounts of G-CSF upon G-CSF activation with LPS. Furthermore, differences in IL-4 and IL-13 were not seen by Luminex array.

### 3.3. G-CSFR Modulates STAT Signaling in Macrophages

**Figure 3:**
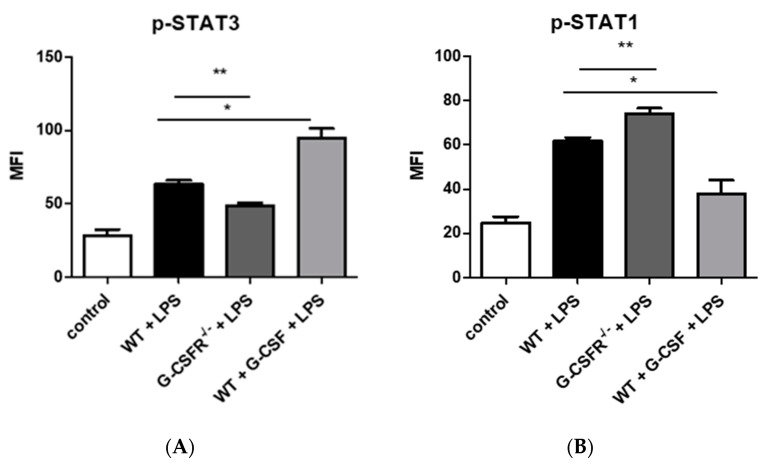
Stat signaling differs between untreated control and LPS-activated WT, G-CSFR^−/−^, and WT + G-CSF BMM as shown by (**A**) p-STAT3 (**B**) and p-STAT1 mean fluorescence intensity (MFI) by cell signaling bead array. *n* = 8; * *p* and ** *p* ≤ 0.05.

It is well established in the literature that TAMs can develop distinct phenotypes with both anti- and pro-tumoral properties. However, those phenotypes exhibit a plasticity, which is heavily influenced by the TME and the cytokine milieu. STAT signaling is considered a major intrinsic pathway implicated in inflammation, proliferation, motility, immune tolerance, angiogenesis, apoptosis, and cancer [24]. More specifically, STAT1 was upregulated in M1 macrophages; thus, it is associated with anti-tumoral activity, while STAT3 is considered a pro-tumorigenic factor and is mainly upregulated in M2 macrophages [25]. We therefore examined the effect of macrophage activation on the levels of phosphorylated STAT1 and STAT3 transcription factors in WT and G-CSFR^−/−^ BMMs. Our data showed that LPS activation generated statistically significant lower levels of p-STAT3 in G-CSFR^−/−^compared to WT cells (Figure 3A). Furthermore, LPS and G-CSF treatment demonstrated even higher levels of p-STAT3, suggesting an M2 phenotype. Concurrently, the opposite effect was observed for the p-STAT1 activity. LPS-activated G-CSFR^−/−^ BMMs exhibited statistically higher levels of the p-STAT1. Co-activation of LPS of WT BMMs with G-CSF led to a reduction in the levels of p-STAT1 (Figure 3B). The above data suggest that G-CSFR^−/−^ BMMs present a phenotype closely related to M1 macrophage subtype consistent with anti-tumoral effects, while the presence of G-CSF/G-CSFR may drive WT macrophages toward a pro-tumoral M2 phenotype. 

### 3.4. Adoptive Transfer of GCSFR^−/−^ BMMs Reduces Tumor Growth

**Figure 4:**
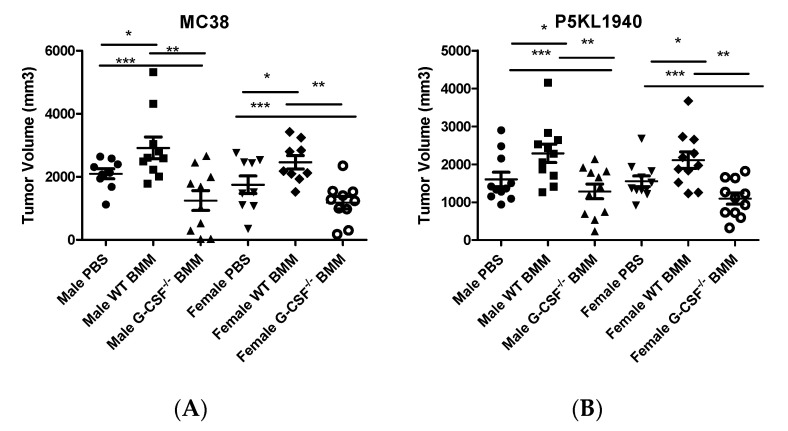
Adoptive transfer of WT or G-CSFR^−/−^ BMMs in (**A**) MC38 or (**B**) PK5L1940 tumor-bearing mice. In both tumor models, injection with G-CSFR^−/−^ macrophages significantly reduces tumor volume in male and female mice. *n* = 10; * *p*, ** *p*, and *p* *** ≤ 0.05 in comparing different mouse groups.

Collectively, the previous results reveal an important role of the G-CSFR in tumor macrophage cytokine production. Thus, we designed experiments to adoptively transfer WT and G-CSFR^−/−^ BMMs in colon (MC38) and pancreatic (PK5L1940) tumors based on a previously published approach [22]. WT or G-CSFR^−^^/−^ BMMs were administered peritumorally on days 1 and 7 after tumor cell injection when tumors were under 500 mm^3^. Tumors were dissected at day 21. At day 21, an approximately 3-fold increase in F4/80 gene expression was detected in tumors that had adoptive transfer of WT or G-CSR^−/−^ BMM compared to control tumors suggesting transferred macrophages remain in the tumor throughout the experiment (Figure S2). Analysis of tumor volume in both tumor types revealed that adoptive transfer of G-CSFR^−/−^ BMMs led to reduced tumor progression and smaller tumor volumes compared to WT BMMs in both MC38 and PK5L1940 (Figure 4A,B). No differences were observed in male vs. female mice, except for slightly larger tumors in male mice receiving WT BMMs. Thus, these data supported our initial hypothesis of the anti-tumoral effects of GCSFR deficiency in macrophages in the tumor microenvironment.

### 3.5. Adoptive Transfer of G-CSFR^−/−^ BMMs Affects the Tumor Microenvironment, Leading to Altered Cytokine Expression

**Figure 5:**
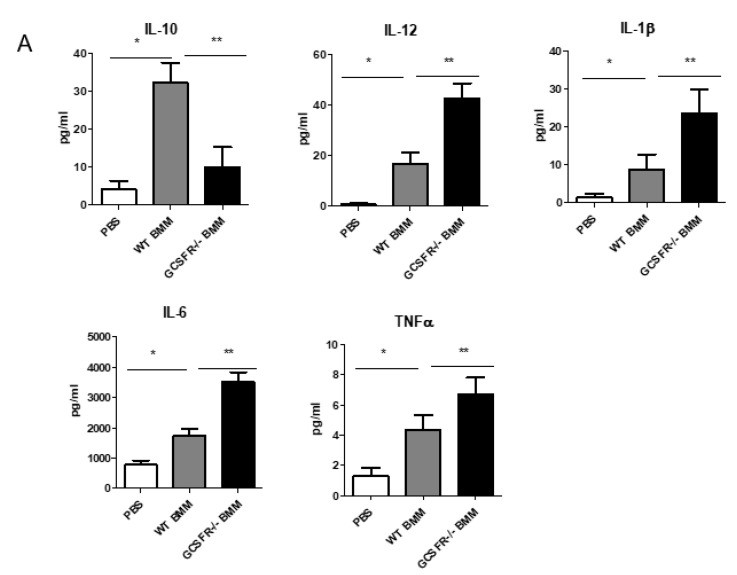

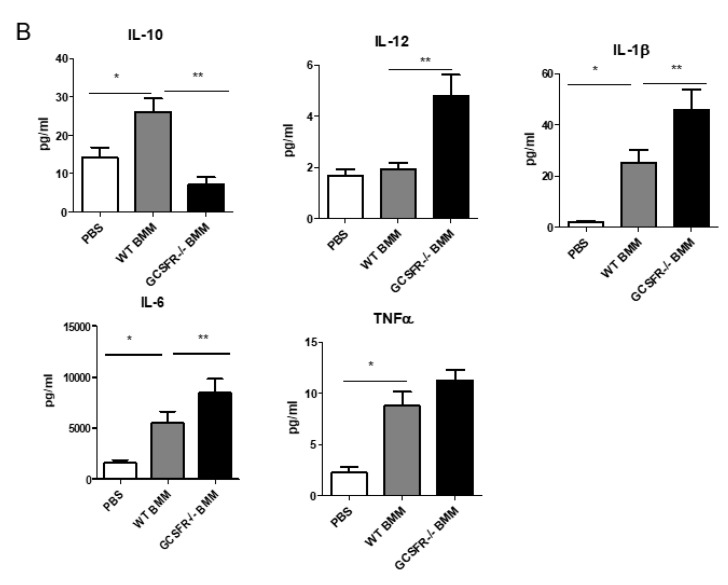
Tumor supernatant cytokine levels differ between control (PBS injections), WT BMM adoptive transfer and G-CSFR^−/−^ BMM adoptive transfer mice in (**A**) MC38 (**B**) PK5L1940 tumors as shown by multiplex bead array. *n* = 10; *p and ** *p* = < 0.05.

To obtain a global view on the cytokine levels upon adoptive transfer, we next examined mouse tumor supernatant cytokines. We found altered cytokine levels in G-CSFR^−/−^ BMM-injected tumors (MC38 and PK5L1940) compared to WT (Figure 5A,B). Results from male and female mice were combined upon finding no significant differences between the sexes in these models. Adoptive transfer of G-CSFR^−/−^ BMMs led to significantly increased levels of proinflammatory cytokines IL-12, IL-1β, IL-6, and TNF-α. Conversely, the levels of the pro-tumoral cytokine IL-10 were significantly reduced with G-CSFR^−/−^ BMMs adoptive transfer (Figure 5A,B). Taken together, the above data indicate an important functional role for G-CSFR in macrophage function in the TME and suggest a possible mechanism through which G-CSFR signaling can control tumor growth.

### 3.6. Ablation of G-CSFR Leads to Increased Levels of Nitric Oxide Production and Apoptosis Marker

**Figure 6:**
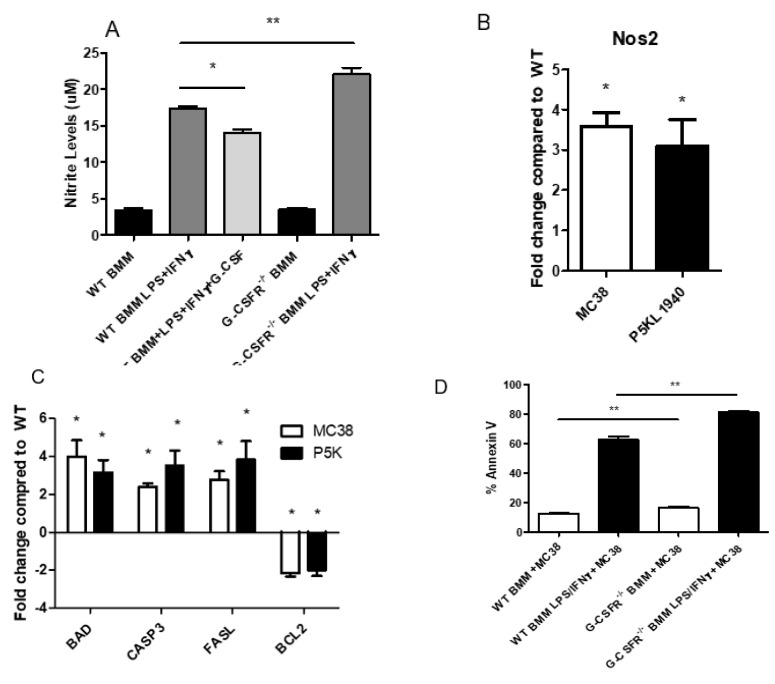
G-CSFR knockdown in macrophages promotes (**A**) increased nitrite levels by fluorometric plate assay, and in adoptive transfer models, (**B**) an increased *NOS2* gene expression, and (**C**) increased gene expression of *BAD*, *CASP3*, and *FASL* apoptosis genes with simultaneous decreased *BCL2* proliferation gene. (**D**) MC38 incubation with LPS- and IFNγ-treated macrophages also shows increased tumor killing by G-CSFR^−/−^ macrophages compared to WT by Annexin V staining for flow cytometry. *n* = 6 for (**A**), 10 for (**B**) and (**C**), and 6 for (**D**). * *p* and ** *p* ≤ 0.05.

Nitric oxide (NO), a key signaling molecule synthesized by NOS2, is involved in several physiological processes. Elevated levels of NO have been directly implicated in pathological conditions, such as chronic inflammation, metabolic diseases, and cancer [26]. The TME is affected by levels of NO in response to growth factors and inflammatory mediators. As established in the literature, high levels of ΝO from activated macrophages can mediate cytotoxicity [27]. Based on these observations, we next examined the NO levels in BMMs. A fluorometric assay was used to identify the NO production in G-CSFR^−/−^ and WT BMM upon co-activation with LPS and IFNγ. Our analysis indicated that WT BMMs produced high levels of NO upon activation as shown in Figure 6A. The addition of recombinant G-CSF to activated WT BMMs led to a significant reduction of NO levels. The activation of G-CSFR^−/−^ BMMs led to increased levels of NO production compared to WT BMMs (Figure 6A). Given that NO levels were increased in G-CSFR^−/−^ BMMs, gene expression of *NOS2* in MC38 and PK5L1940 tumors with BMM adoptive transfers were determined. *NOS2* gene expression showed 3.5- and 3-fold changes in MC38 and PK5L1940 tumors adoptively transferred with BMMs respectively, when compared with the WT BMMs (Figure 6B). We next examined the potential impact of these observations in our adoptive transfer experiments on apoptosis. Increased NO levels are linked to upregulation of Fas/FasL, leading to tumor killing through activation of the apoptotic machinery [28]. In order to answer this question, we analyzed several apoptosis markers, such as *BAD, CASP3, FASL*, and *BCL2* by qPCR analysis in the MC38 and PK5L1940 tumors. Our analyses demonstrated that both tumors presented higher expression levels of pro-apoptotic genes *BAD*, *CASP3, FASL*, and lower gene expression levels of the anti-apoptotic *BCL2* after injection with G-CSFR^−/−^ BMMs compared to WT BMMs (Figure 6C). Finally, to directly measure the tumor killing effects of BMMs on tumor cells, we performed an in vitro tumor killing assay in co-cultured BMMs with MC38 tumor cells. Our results showed that activated and non-activated G-CSFR^−/−^ BMMs produced significantly higher killing of tumor cells compared to WT BMMs (Figure 6D). All the above data conclude that lack of G-CSFR boosts macrophage cytotoxicity and leads to tumor cell destruction.

## 4. Discussion 

Several studies have documented cross talk between cancer and immune cells, but also inflammatory factors that are expressed by the TME, such as the CSF family of cytokines, which attract macrophages (through CCL2 and VEGFA) [29,30,31,32]. We have shown that G-CSF is highly expressed in GI cancers [17,33] and is associated with tumor progression and poor prognosis in several other cancer types, such as prostate [34], neuroblastoma [35], bladder [36], non-small cell lung cancer [37], and head and neck squamous cell carcinomas [38]. However, little is known regarding the role of the G-CSF in the TME, particularly related to immune cells. Our lab has previously demonstrated how the genetic ablation of the G-CSFR can modulate the innate immune response in colon and pancreatic cancer directly modulating T cell responses and tumor growth [18]. In the current study, we examined the role of G-CSFR in macrophages in colon and pancreas tumors. 

Our results showed that gene expression levels of *IL10* and *ARG1* were downregulated, while *IL-12* and *NOS2* were upregulated in *in vitro*-differentiated BMM lacking G-CSFR. The *IL-10* and *IL-12* results were also confirmed by multiplex cytokine analysis, where reduced levels of IL-10 and increased levels of IL-12 cytokine production in supernatants of activated G-CSFR^−/−^ BMM were determined. These observations are in line with studies showing elevated levels of these specific cytokines upon tumor progression. In humans, IL-10 serum levels increase over time during CRC progression, correlating with poor prognosis [39,40,41,42], while high levels of ARG-1 were associated with stage III–IV CRC tumors and lymph node metastasis [43]. Conversely, the protective roles of IL-12 and NOS2 have also been documented. Administration of IL-12 serves as a potential tumor-suppressive treatment as it has the ability to collaborate with other cytokines (such as GM-CSF), providing immunomodulatory effects on the TME [44]. On the other hand, NOS2 has been implicated in macrophage-mediated anti-tumoral activity in various human tumors through inhibition of tumor growth and metastasis [45]. Furthermore, the anti-tumoral effect of NOS2 was also demonstrated in mice, where exposure to NO reduced the incidence of colitis-induced adenocarcinoma, through regulation of abnormal activity of the p53 pathway [46]. We also showed that two more cytokines, IL-1β and TNF-α were detected in high levels in G-CSFR^−/−^ BMMs, while in activated WT BMMs treated with exogenous G-CSF, a dramatically decreased production of those cytokines was observed. These cytokines may have both pro- and anti- tumoral effects, but are known to be produced by M1 macrophages [47]. Another explanation for these cytokines may be that those pro-inflammatory cytokines are secreted by macrophages and may initiate adaptive immune responses through the polarization of CD4^+^ T cells towards Th1 phenotypes, thus exhibiting anti-tumor activity.

STATs are considered a transcription factor that influences macrophage phenotypes and their response in the TME and are directly linked with tumorigenesis and tumor progression. Two important members of the STAT family that play opposing roles in tumorigenesis by regulating macrophage phenotype and cytokine production are STAT1 and STAT3. Our results showed that upon LPS activation, GCSFR^−/−^ BMMs displayed increased levels of phosphorylated STAT1, with a corresponding reduction in phosphorylated STAT3. Other studies have demonstrated that STAT1 activation is upregulated in M1 macrophages and is correlated with developing antitumor responses, while STAT3 is considered an oncogene promoting tumor growth. Moreover, in the tumor microenvironment, STAT3 activation in TAMs can lead to inhibition of IL-12 through NFκB pathway blockade [48]. All the in vitro experiments presented in this study showed that the GCSFR^−/−^ BMMs demonstrated a phenotype closely associated with anti-tumoral functions and furthermore that the addition of exogenous G-CSF in WT BMMs led to the development of pro-tumoral characteristics. 

TAMs consist of a heterogeneous group of cells that are not molecularly and immunologically well characterized. M1 macrophages produce numerous pro-inflammatory cytokines such as IL-12, IL-18, CXCL8, and CXCL13, while M2 macrophages produce anti-inflammatory cytokines such as IL-4, IL-10, IL-13, TGF-β, and Arginase-1 [49]. Although macrophage polarization is a dynamic process, M1 and M2 phenotypes do not represent terminally differentiated macrophages, but are often characterized by plasticity or “re-education”, where the M1/M2 ratio can adapt to the tumor microenvironment and an ever-changing tumor based-cytokine milieu [50]. Even though in several types of cancer, such as bladder cancer, breast, melanoma, and kidney, TAMs are associated with an M2 phenotype, in CRC patients, an increase in TAMs correlates with a better prognosis [51]. The observed anti-tumoral phenotype of the G-CSFR^−/−^ BMMs from the in vitro experiments was also maintained during the adoptive cell transfer experiments, where the mice that were injected with the G-CSFR^−/−^ BMMs demonstrated reduced tumor growth, in both pancreatic and colorectal mouse transfer models. Furthermore, cytokine profiling of the adoptively transferred tumors confirmed that G-CSFR^−/−^ macrophages exhibit anti-tumoral identity. This could also point to a correlation and a cross talk between adaptive (T cells) and innate (macrophages) immune system responses in the struggle to control the tumor progression. 

Finally, we also showed that genetic deletion of G-CSFR led to increased nitric oxide and NOS2 production and apoptosis markers. These markers in macrophages are directly linked to high levels of cytotoxicity. In tumors, macrophages can orchestrate cell death and apoptosis [52]. Tumoricidal macrophages are critical in promoting tumor killing and apoptosis through the release of cytotoxic factors and also enhance the production of pro inflammatory cytokines, such as IL-1β and TNF-α. Based on these data and our results, we suggest that in the absence of G-CSFR, tumor cytotoxicity is amplified, leading to the creation of a tumor microenvironment favorable to the prevalence of the anti-tumor macrophages. 

For the past decades, a plethora of studies have attempted to elucidate the cross talk between heterogeneous tumor cells and the tumor microenvironment, in order to provide better answers to our understanding of the cancer progression and strategies for cancer treatment. Our past work has provided evidence that tumors with high expression of G-CSFR are resistant to immunomodulatory strategies, which pinpoints the beneficial functions of inhibiting G-CSFR in tumor therapy. Promising findings regarding the crucial role of the G-CSFR in GI cancers and a better understanding of the molecular tumor immune escape mechanisms may in the future open new avenues for the development of clinically relevant applications.

## 5. Conclusions

The results presented here indicate that G-CSF/G-CSFR regulate macrophage function in the context of GI tumors. Decreased IL-10 and ARG1 levels and increased IL-12 and NOS2 levels, along with changes in other proinflammatory cytokines, in G-CSFR-deficient BMM, suggest G-CSFR promotes an overall pro-tumorigenic immune phenotype. Concomitant changes in tumor size, apoptosis markers, tumor killing, and cytokine production in tumors injected with BMM were consistent with in vitro changes in IL-10, IL-12, and other proinflammatory cytokines. Taken together, our data indicate that G-CSF/G-CSFR play an important role in regulating macrophage function and suggest possible strategies in cancer therapeutics.

## Supplementary Materials

The following are available online at https://www.mdpi.com/2072-6694/12/10/2868/s1, Figure S1: BMM cells express (A) F4/80 and (B) G-CSFR in the CD115 negative population as shown by flow cytometry of in vitro differentiated BMM, Figure S2: MC38 and P5KL1940 tumors have increased F4/80 gene expression compared to control tumors indicating approximately 3-fold increase in the macrophage marker for both WT and G-CSFR^−/−^ adoptive transfer experiments. *n* = 6, * *p* > 0.05.

## Abbreviations

CRC	Colorectal cancer
G-CSF	Granulocyte colony-stimulating factor
BMM	Bone marrow derived macrophage
TAM	Tumor-associated macrophage
TME	Tumor microenvironment
NO	Nitric oxide
NOS2	Nitric oxide synthase 2
LPS	Lipopolysaccharide
TNFα	Tumor necrosis factor alpha
ROS	Reactive oxygen species
STAT	Signal transducer and activator of transcription
FasL	FAS ligand
