# Supplementary material for: G-CSF and G-CSFR Induce a Pro-Tumorigenic Macrophage Phenotype to Promote Colon and Pancreas Tumor Growth
Source: Cancers (Basel). 2020 Oct 6;12(10):2868. doi: 10.3390/cancers12102868 (PMC7601499; doi:10.3390/cancers12102868)
Supplement: Supplementary file 1 [file cancers-12-02868-s001.pdf]

cancers

MDPI

*Supplementary Materials*

# G-CSF and G-CSFR Induce a Pro-Tumorigenic Macrophage Phenotype to Promote Colon and Pancreas Tumor Growth

**Ioannis Karagiannidis, Eliane de Santana Van Vilet, Erika Said Abu Egal, Brandon Phinney, Damian Jacenik Eric R. Prossnitz and Ellen J. Beswick**

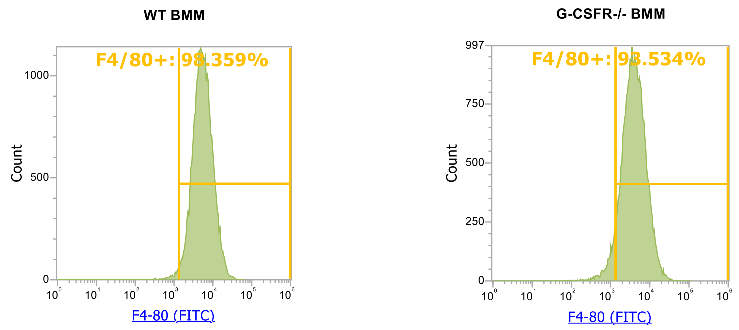


(**A**)

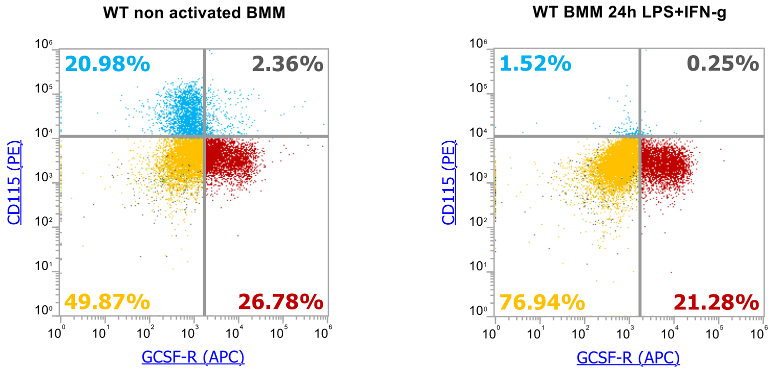


(**B**)

**Figure S1.** BMM cells express (**A**) F4/80 and (**B**) G-CSFR in the CD115 negative population as shown by flow cytometry of in vitro differentiated BMM.

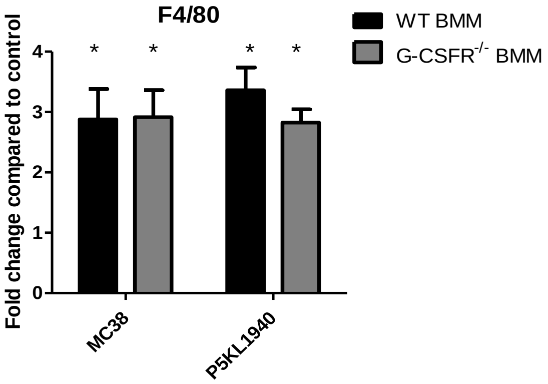


**Figure S2.** MC38 and P5KL1940 tumors have increased F4/80 gene expression compared to control tumors indicating approximately 3-fold increase in the macrophage marker for both WT and G-CSFR$^{-/-}$ adoptive transfer experiments. $n = 6$, * $p > 0.05$.

© 2020 by the authors. Licensee MDPI, Basel, Switzerland. This article is an open access article distributed under the terms and conditions of the Creative Commons Attribution (CC BY) license (http://creativecommons.org/licenses/by/4.0/).
